# The optimal sequence of radiotherapy and chemotherapy in adjuvant treatment of breast cancer

**DOI:** 10.1186/1755-7682-4-35

**Published:** 2011-10-16

**Authors:** Hamza Abbas, Ashraf Elyamany, Mohamed Salem, Ahmed Salem, Salah Binziad, Basem Gamal

**Affiliations:** 1 Department of Radiation Oncology, South Egypt Cancer Institute, Assiut University, Egypt; 2 Department of Medical Oncology, South Egypt Cancer Institute, Assiut University, Egypt; 3 Department of surgical Oncology, South Egypt Cancer Institute, Assiut University, Egypt

**Keywords:** breast cancer, chemotherapy, radiotherapy, sequence

## Abstract

**Background:**

The optimal time sequences for chemotherapy and radiation therapy after breast surgery for patients with breast cancer remains unknown. Most of published studies were done for early breast cancer patients. However, in Egypt advanced stages were the common presentation. This retrospective analysis aimed to assess the optimum sequence for our population.

**Methods:**

267 eligible patients planned to receive adjuvant chemotherapy [FAC] and radiotherapy. Majority of patients (87.6%) underwent modified radical mastectomy while, 12.4% had conservative surgery.

We divided the patients into 3 groups according to the sequence of chemotherapy and radiotherapy. Sixty-seven patients (25.1%) received postoperative radiotherapy before chemotherapy [group A]. One hundred and fifty patients (56.2%) were treated in a sandwich scheme (group B), which means that 3 chemotherapy cycles were given prior to radiotherapy followed by 3 further chemotherapy cycles. A group of 50 patients (18.7%) was treated sequentially (group C), which means that radiotherapy was supplied after finishing the last chemotherapy cycle. Patients' characteristics are balanced between different groups.

**Results:**

Disease free survival was estimated at 2.5 years, and it was 83.5%, 82.3% and 80% for patient receiving radiation before chemotherapy [group A], sandwich [group B] and after finishing chemotherapy [group C] respectively (p > 0.5). Grade 2 pneumonitis, which necessitates treatment with steroid, was detected in 3.4% of our patients, while grade 2 radiation dermatitis was 17.6%. There are no clinical significant differences between different groups regarded pulmonary or skin toxicities.

**Conclusion:**

Regarding disease free survival and treatment toxicities, in our study, we did not find any significant difference between the different radiotherapy and chemotherapy sequences.

## Background

Adjuvant chemotherapy and radiotherapy are established treatment of breast cancer. The optimal way to integrate chemotherapy and radiation therapy after breast surgery for patients with breast cancer remains unknown [1]. Generally, radiotherapy is used after completion of adjuvant chemotherapy [2] but decisive data for a scientifically based decision on the optimal sequence are not known.

Retrospective reviews have shown increased rates of local-regional recurrence when radiation therapy is delayed after surgery [3]. This has not been a uniform finding, however, with other retrospective series reporting no increased risk of local recurrence when radiation is delayed in order to administer chemotherapy [4-6]. There is also concern that delaying chemotherapy in order to give radiation may increase the risk of distant metastasis and ultimately affect survival [7,8]. In Egypt, patients usually presented with advanced stage of cancer and majority of patients underwent modified radical mastectomy, which is different from patients' characteristic in other countries. We conducted this retrospective analysis to evaluate the optimum sequence of radiotherapy and chemotherapy among Egyptian patients.

## Methods

The present retrospective study was conducted on 267 breast cancer patients attending to South Egypt Cancer institute, Assiut University over the period from January 2001 to June 2008. Eligible criteria for the retrospective analysis were Patients ≥ 35 years of age, female gender, performance status ≤ II according to ECOG scale, histological evidence of invasive breast cancer, non-metastatic breast cancer planned to receive adjuvant chemotherapy [FAC] and radiotherapy.

Surgery was done including either modified radical mastectomy or breast conservative surgery.

Chemotherapy regime was six cycles of anthracycline-based chemotherapy (FAC) were given to all patients. FAC (5-fluorouracil 500 mg/m^2^, adriamycin 50 mg/m^2^, and cyclophosphamide 500 mg/m^2^) were given bolus intravenous day one every 3 weeks.

Our Radiation target volume was chest wall, breast (in case of breast conservative surgery) and supraclavicular region if indicated. The supraclavicular region extended from the level of cricothyroid groove to sternal angle. The superior border of the chest wall and breast matched with the lower border of the supraclavicular field. The lower field border was at 1 cm below the mammary fold, guided by the opposite breast, if mastectomy had been performed. The medial border was set at midline, with no specific attempt to cover the internal mammary lymph nodes, while the lateral border was set at mid-axillary line. In patient who underwent modified radical mastectomy, we used 2D planning however, and we used 3D planning for patient underwent breast conservative surgery. A complete 3D plan was done, taken in consideration the ICRU 50 recommendations (A certain degree of heterogeneity should be kept within +7% and -5% of prescribed dose).

Daily fractions of 2Gy were given to both supraclavicular (at 3 cm depth) and tangential chest fields (at depth of isocenter), with a total dose of 50 Gy with 6MV photon using Linac (Siemens Mevatron). Patients who had undergone conservative surgery received boost dose to tumour site of 14Gy (2Gy daily fractions) with electron beam of 9-12 MeV prescribed at the 90% isodose depth, at the end of whole breast irradiation.

The patients were grouped according to the sequence of chemotherapy and radiotherapy. Sixty - seven patients (25.1%) were treated with radiotherapy postoperative before chemotherapy [group A]. One hundred and fifty patients (56.2%) were treated in a sandwich scheme (group B) which means that 3 chemotherapy cycles were given prior to radiotherapy followed by 3 further chemotherapy cycles. A group of 50 patients (18.7%) were treated sequentially (group C) which means that radiotherapy was applied after finishing the last chemotherapy cycle.

The observation period started at the time of surgery with median follow up was 30 months. The distribution of various categorical variables, in the total patients as well as separately for the subgroups of A, B and C is summarized in Table 1.

**Table 1:** patients' characteristics

Variable	All patients [%]	Group A [%]	Group B [%]	Group C [%]	p
**Total number**	267	67	150	50	

**Age at diagnosis (Mean) year**	49.5	50	49	50.3	0.9

**Menopausal status**					
**Premenopausal**	108 [40.7]	26 [38.8]	62 [41.3]	20 [40]	p > 0.5
**perimenopausal**	48 [18]	15 [22.4]	25 [16.7]	8 [16]	
**postmenopausal**	111 [41.3]	26 [38.8]	63 [42]	22 [44]	

**Laterality**					p > 0.5
**Right**	116 [56.6]	32 [47.8]	60 [40]	24 [48]	
**Left**	151 [43.4]	35 [55.2]	90 [60]	26 [52]	

**Stage**					p > 0.5
**I**	18 [6.7]	5 [7.5]	9 [6]	4 [8]	
**II**	181 [67.8	39 [58.2]	109 [72.7]	33 [66]	
**III**	68 [25.5]	23 [34.3]	32 [21.3]	13 [26]	

**Type of surgery**					p > 0.5
**MRM**	234 [87.6]	57 [85.1]	131 [87.3]	43 [86]	
**BCS**	33 [12.4]	10 [14.9]	19 [12.7]	7 [14]	

**Hormonal receptor**	151 [56.6]	41 [61.2]	79 [52.6]	31 [14]	p > 0.5
**Positive**	116 [43.4]	26 [38.8]	71 [47.4]	19 [12]	
**Negative**					

We reported grade 2 toxicity or higher; moderate pulmonary toxicity means respiratory symptoms judged by the clinician to be caused by radiotherapy and treated with corticosteroids [9]. Grade 2 Skin toxicity defined as moderate to brisk erythema or a patchy moist desquamation, mostly confined to skin folds and creases; moderate edema [10].

## Statistical methods

Continuous variables were summarized by means. Categorical data were condensed by absolute and relative values. Cross tabulations were created to compare frequency distributions between subgroups. The Pearson × 2-test was used to assess whether the associations displayed in those cross tabulations are statistically significant. Disease free survival (DFS) curves were estimated according to the Kaplan-Meier method. The log-rank test was used for comparison of DFS-curves between the subgroups according to sequencing of chemo- and radiotherapy.

The global significance level for all statistical test procedures conducted was chosen as = 5%. All statistical analyses were conducted in an explorative manner. Thus, with consideration of the explorative character of the analysis, p-values of p = 0.05 can be interpreted as statistically significant test results.

## Results

All patients' characteristics were summarized in Table 1; from these data, we can report that median age among our patients was 49.5 years. Premenopausal, perimenopausal and postmenopausal women represented 40.7% (108 patients), 18% (48 patients) and 41.3% (111 patients) respectively. Right sided breast cancer represent 56.6% (116 patients), while 43.4% (151 patients) had left side.

Most of patients (181 patients) were stage II (67.8%), stage I represent 6.7% (18 patients) while, sixty-eight patients (25.5%) had stage III.

Majority of the patients (234 patients) which constituted 87.6% underwent modified radical mastectomy (MRM) while, thirty-three patients (12.4%) had breast conservative surgery (BCS).

The differences in patients' characteristics among different groups of radiotherapy and chemotherapy sequence are represented in Table 1. The Pearson x^2^-test was used to assess these differences and revealed no clinical significant differences between all patient characteristic (p > 0.5) among different group.

Among our patients, we noticed that although the incidence of respiratory symptoms was high there were mild and self-limiting with most of cases showed resolution within 12 months. Grade 2 pneumonitis, which necessitates treatment with steroid, between our patients detected in 3.4%, while grade 2 radiation dermatitis was 17.6%. There are no clinical significant differences between different groups regarded pulmonary or skin toxicities (Table 2). Cardiac toxicities were very low and not more than G1.

**Table 2:** Treatment related toxicities

Variable	All patients [%]	Group A [%]	Group B [%]	Group C [%]	p
**pulmonary complication GII**	9 [3.4]	3 [4.5]	4 [2.7]	2 [4]	> 0.05

**Skin toxicity GII**	47 [17.6]	16 [24]	21 [14]	10 [20]	> 0.05

**Figure 1:**
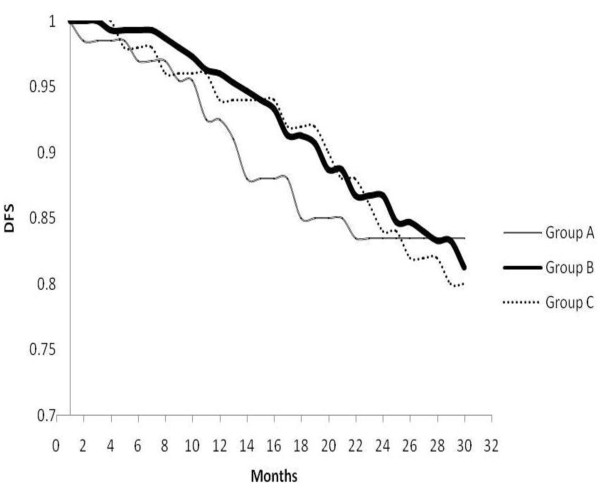
**Disease free survival of different groups**.

As shown in Table 3 disease free survival (DFS) was estimated at 2.5 years, and it was 83.5%, 82.3% and 80% for patient receiving radiation before chemotherapy [group A], sandwich [group B] and after finishing chemotherapy [group C] respectively, by applying Pearson x2-test, and the differences was insignificant (p > 0.5). Figure 1 demonstrate the Kaplan-Meier curves for disease free survival for the three groups, using log-rank test, we did not find any significant differences between different groups (p > 0.5).

**Table 3:** 2.5 years DFS among each group

Group A	Group B	Group C	P-value
83.5	81.3	80	0.90

## Discussion

Chemotherapy and radiation therapy are typically not given concurrently in patients with breast cancer because of the widespread use of anthracycline-based chemotherapy regimens and the concern for excessive radiation toxicity with concurrent treatment. Hence, it is necessary to decide how best to sequence systemic and radiation therapies. This question arises for both patients treated with lumpectomy and those treated with mastectomy, however, the optimal sequencing of adjuvant chemotherapy and radiotherapy in breast cancer patients remains controversial [11].

Many studies suggested that delaying the initiation of radiotherapy might result in an increased likelihood of local failure. Buchholz and his colleagues divided 105 patients with local-regional breast cancer into two groups based on the timing of their radiation treatments; early radiation group [patients began their radiation within 6 months of their diagnosis] and delayed radiation group [patients began their radiation after 6 months of their diagnosis]. They concluded that, delay in the initiation of radiation for a period of 6 months or greater from diagnosis resulted in a higher local failure rate. Furthermore, this higher local failure rate was associated with an increased rate of distant metastases and a decreased overall survival rate [12].

Hartsell and his colleagues studied the impact of delaying irradiation to the intact breast on 474 patients underwent lumpectomy and intact breast irradiation for early stage invasive breast cancer. Chemotherapy was administered to 84 patients with median follow-up was 62 months. They concluded that delays in the initiation of irradiation are associated with increased risk of relapse in the breast. When possible, the interval between definitive breast surgery (lumpectomy or re-excision) and the initiation of radiation therapy should be fewer than 120 days [13]. Recht and his colleagues randomized 244 patients with clinical stage I or II breast carcinoma after surgery to receive chemotherapy either before or after radiotherapy. they suggested that, it is preferable to give 12 weeks of chemotherapy before irradiation, rather than radiotherapy first, to patients at substantial risk for systemic recurrence of cancer. Although their results suggest that the effect of the delay in initiating chemotherapy may be greatest for patients with the highest risk of subclinical systemic disease (i.e., those with four or more positive nodes) and that the delay in initiating radiotherapy may be most detrimental to patients with close or positive margins of the resected tumour. In addition, extrapolating the results of this trial to other regimens, particularly those with more prolonged intervals between surgery and radiotherapy (e.g., six months or more), may be misleading [14]. Update of this trail with 135 months median follow-up for surviving patients, there were no significant differences between the Chemotherapy-first and RT-first arms in time to any event, distant metastasis, or death. Sites of first failure were also not significantly different. However, this study has several limitations. The statistical power of their subgroup analyses is low and subgroup analyses must be viewed with special caution [15]. In addition, many studies suggest that delaying the initiation of radiotherapy may result in an increased likelihood of local failure [16].

Buchholz and his colleagues conducted a retrospective analysis of 124 patients with lymph node-negative breast cancer, underwent breast-conserving surgery with axillary dissection, followed by chemotherapy and radiation therapy. The outcome of 68 patients who received chemotherapy first was compared with that of 56 patients who received radiation first. There were no statistically significant differences in local control, disease-free survival, or overall survival between the two groups. They concluded that chemotherapy can be giving before radiation in lymph node-negative breast cancer without compromising local control. Given the concerns about increased distant metastases if radiation is given first, the chemotherapy-radiation sequence is recommended [17].

Contrary to above results, Leonard and his colleagues failed to identify any surgery-radiotherapy interval that resulted in increased local recurrence if radiotherapy was delayed for administration of adjuvant chemotherapy in breast cancer patients. They studied the records of 262 women with 264 cases of breast cancer. Group I contained 105 patients treated with conservative surgery, chemotherapy, and radiotherapy. Group II contained 157 patients (used as a concurrent control) treated with conservative surgery and radiotherapy only. There were no significant differences in local recurrence in any surgery-radiotherapy interval within each group. However, this failure may be due to the heterogeneous population of breast cancer patients, and because group II did not receive chemotherapy [6].

A retrospective analysis aimed to assess the role of sequencing in patients after mastectomy was conducted. They studied records of a total of 212 patients. Eligible patients had a stage III breast cancer and received adjuvant chemotherapy and radiotherapy after mastectomy and axillary dissection. Eighty-six patients were treated sequentially (chemotherapy followed by radiotherapy) (SEQ-group), 70 patients had a sandwich treatment (SW-group) and 56 patients had simultaneous chemo-radiation (SIM-group). 5-year overall- and disease free survival were 53.2%/56%, 38.1%/32% and 64.2%/50%, for the sequential, sandwich and simultaneous regime, respectively, which differed significantly in the univariate analysis (p = 0.04 and p = 0.03). The 5-year locoregional or distant recurrence free survival showed no significant differences according to the sequence of chemo- and radiotherapy. They concluded that, no clear advantage can be stated for any radio- and chemotherapy sequence in breast cancer therapy so far [1].

National comprehensive cancer network panel [2] recommended that radiotherapy should be started after finishing chemotherapy however; this is based on single prospective trail [14] and its update [15] with limitations as mentioned above.

French multicenter phase III randomized trial (ARCOSEIN trial) enrolled 716 patients. Sequential treatment of Chemotherapy administered first followed by RT was compared with concurrent treatment of Chemotherapy administered with RT. The Chemotherapy regimen consisted of mitoxantrone (12 mg/m2), fluorouracil (500 mg/m2), and cyclophosphamide (500 mg/m2) on day 1, which was repeated every 21 days for six courses. RT was delivered to the breast and, when indicated, to the regional lymphatics. There was no statistically significant difference on 5-year DFS, locoregional recurrence-free survival, metastasis-free survival, or overall survival. Nevertheless, in the node-positive subgroup, the 5-year LRFS was statistically better in the concurrent arm (97% in concurrent v 91% in sequential; P = .02), corresponding to a risk of locoregional recurrence decreased by 39% (hazard ratio, 0.61; 95% CI, 0.38 to 0.93). They concluded that, this treatment protocol remains an appealing clinical option for patients at a high risk of recurrence [18]. Ismaili et al evaluated the efficacy and safety of the concomitant use of anthracycline with radiotherapy after mastectomy or BCS. The adjuvant treatment, based on anthracycline and concurrent RT, reduced breast cancer relapse rate, and significantly improved LRFS, EFS and OS in patients receiving more than 1 cycle of concurrent Chemotherapy. There were more hematologic and non hematologic toxicities in the anthracycline group compared to those received CMF [19].

Zellars RC etal conducted a single-arm feasibility trial testing anthracycline-based chemotherapy and concurrent partial breast irradiation (PBI). They concluded that PBI with concurrent dose-dense doxorubicin and cyclophosphamide (ddAC) is feasible with acceptable local and systemic toxicity [20].

The different treatment sequences at our department were related to changing our department policies as a part of radiation therapy evolution. Our treatment protocols arranged for immediate postoperative radiation before chemotherapy. However after June 2002, we established sandwich scheme which means that 3 chemotherapy cycles were given prior to radiotherapy followed by 3 further chemotherapy cycles. Sandwich scheme was based on the data reported by Recht and his colleagues as they noted that delaying breast irradiation longer than 16 weeks after tumour excision resulted in a higher incidence of breast relapses. While Administration of irradiation first led to a higher incidence of distant metastases [21]. With further evolution of radiation protocols, since June 2005, we preferred to finish chemotherapy first before radiation, similar to other cancer centres protocols based on many studies [14,15].

We used three dimensions planning only for BCS, however two dimensions planning is used in-patient underwent mastectomy. This is based in study done at South Egypt Cancer Institute during the period of time from February 2001 to October 2003. Its results is published in 2004 and demonstrated that there is significant dosimetric improvement from two dimensions planning to three dimensions planning in both patient underwent mastectomy and patient underwent BCS. However, this improvement is marked in BCS that is reflected by decrease skin toxicity [22].

Moderate radiation pneumonitis, which necessitates treatment with steroid, between our patients was detected in 3.4% of patients. This result matches with Lingos [23] and Elsayed [22], as they reported incidences of radiation pneumonitis that required steroid 2.9% and 2.7% respectively. Lind and his colleagues [24] reported that 9% of patients had radiation pneumonitis that required steroid. This difference explained by three reasons, the first is that 95% of patients received internal mammary irradiation, the second is that 21% of patients received CMF regimen which contains methotrexate with high tendency to cause pulmonary complications. and the third reason, is higher percentage of irradiated lung volume (32%) in that study that received ≥ 25 Gy. In addition, Hanna and his colleagues [25] reported more incidences, 15% of patients, required steroid for treatment of radiation pneumonitis, and this may be explained by the use of paclitaxol which known to reduce the lung tolerance.

Acute radiation dermatitis (G 2) was detected in 17.6% and this finding is in agreement with other studies [22].

The present study showed a 2.5-year relapse free survival rate was 83.5%, 82.3% and 80% for patient receiving radiation before chemotherapy [group A], sandwich [group B] and after finishing chemotherapy [group C] respectively (p > 0.5). This was similar to that (81%) reported by Ragaz [26] and to that (83.5%) reported by Elsayed [22].

Our study did not find any significant difference in survival or toxicities between the different radiotherapy sequences which is inconsistent with the above mentioned studies however we have many limitation. This is a retrospective study done in different time period as arm (A) was done before June 2002, arm (B) between June 2002 and June 2005, while arm (C) after that; however our patients' characteristics are matched. Follow up period of our patients is short because this study is limited by follow up of last sequence (arm C). The number of cases in arm C is small as many patients received taxane based adjuvant chemotherapy that might be ineligible for our analysis.

Regarding disease free survival or treatment toxicities, our study did not find any significant difference between the different radiotherapy sequences, which is inconsistent with the above-mentioned studies.

We concluded that until now we have no optimal sequence, and it is better to conduct a randomized trial to answer this question.

## Competing interests

The authors declare that they have no competing interests.

## Authors' contributions

all authors have same contributions in collecting data from patients' files. First, second, third and fourth authors underwent statistical analysis. The first author underwent the remaining works (abstract, background, methods, results, discussion, and references). The second author underwent submission of the manuscript. The sixth author did English revision. All authors read and approved the final manuscript.
